# Overview and commentary of the CDEI's extended roadmap to an effective AI assurance ecosystem

**DOI:** 10.3389/frai.2022.932358

**Published:** 2022-08-10

**Authors:** Ethan Barrance, Emre Kazim, Airlie Hilliard, Markus Trengove, Sara Zannone, Adriano Koshiyama

**Affiliations:** ^1^Holistic AI, London, United Kingdom; ^2^Department of Computer Science, University College London, London, United Kingdom; ^3^Institute of Management Studies, Goldsmiths, University of London, London, United Kingdom

**Keywords:** artificial intelligence (AI), roadmap, regulation, standards, compliance, ethics, governance

## Abstract

In recent years, the field of ethical artificial intelligence (AI), or AI ethics, has gained traction and aims to develop guidelines and best practices for the responsible and ethical use of AI across sectors. As part of this, nations have proposed AI strategies, with the UK releasing both national AI and data strategies, as well as a transparency standard. Extending these efforts, the Centre for Data Ethics and Innovation (CDEI) has published an AI Assurance Roadmap, which is the first of its kind and provides guidance on how to manage the risks that come from the use of AI. In this article, we provide an overview of the document's vision for a “mature AI assurance ecosystem” and how the CDEI will work with other organizations for the development of regulation, industry standards, and the creation of AI assurance practitioners. We also provide a commentary of some key themes identified in the CDEI's roadmap in relation to (i) the complexities of building “justified trust”, (ii) the role of research in AI assurance, (iii) the current developments in the AI assurance industry, and (iv) convergence with international regulation.

## Introduction

Artificial intelligence (AI) ethics is a nascent field that has been gaining traction in recent years and aims to address and mitigate the risk of the use of AI through an interdisciplinary approach, drawing on concepts from disciplines including philosophy, computer science, and law (Kazim and Koshiyama, 2021). One of the key themes emerging in this field is the need for algorithm assurance through greater governance, regulation, and standardization of best practices (Kazim and Koshiyama, 2020). Indeed, the term “AI assurance” has garnered popularity with researchers, business leaders, and policymakers alike (Freeman et al., 2022) and can be defined as a process applicable to all stages of AI development to create accountability for the validity, verification, trust, and explainability of AI, as well as promoting an ethical, fair, and unbiased approach (Batarseh et al., 2021). In response to this movement, the UK government has released a series of standards for the use of AI, with the Centre for Data Ethics and Innovation (CDEI), publishing its AI Assurance Roadmap (hereafter “*The Roadmap*”) in December 2021 (CDEI, 2021).

*The Roadmap* is part of a sustained digital/data policy and regulatory agenda by the UK government. Specifically, the then-forthcoming publication of *The Roadmap* was cited in the UK National AI Strategy as being part of its 10-year plan. This joint publication between the Department for Digital, Culture, Media, and Sport (DCMS), who published the National Data Strategy (2020), the Department for Business, Energy, and Industrial Strategy, and the Office for Artificial Intelligence (Kazim et al., 2021; National AI Strategy—HTML version, 2021), is, according to our reading, a signaling document for the vision for innovation and opportunity, underpinned by a trust framework that has innovation and opportunity at its center (Kazim et al., 2021). As such, *The Roadmap* should be read within the broader context of the industrial and legislative agenda of the UK government. More specifically, the past few years have seen a burgeoning in AI governance, and assurance—for example, the CDEI published a blog “the need for AI assurance” in March 2021 (The need for effective AI assurance—Centre for Data Ethics and Innovation Blog, 2021) in which AI assurance is defined, existing models of assurance, and the approach of the CDEI for developing this ecosystem are outlined. *The Roadmap* outlines the CDEI's vision of what a “mature AI assurance ecosystem” would look like, including the introduction of new legislation, stimulus for AI-related education and accreditation, as well as the creation of a professional service for the management and implementation of trustworthy AI systems. The CDEI also highlights how this proposed AI assurance ecosystem will benefit the UK economy. A PricewaterhouseCoopers estimate states that “AI will add 10.3% to UK GDP between 2017 and 2030” (PricewaterhouseCoopers, 2017).

While there are two versions of *The* Roadmap (the ministerial brief and the extended version), the focus of this paper is to review the extended version of *The Roadmap* (CDEI, 2021). We do this primarily by providing an overview of its content, which we do by reproducing the structure of *The Roadmap* itself in Section Overview. Following this, in Section Commentary we offer some commentary on *The Roadmap*, which touches on issues such as accreditation, “ethics washing”, technical standardization, industry developments, and other global AI strategies such as that of the EU (European Commission, 2021), USA (Wyden et al., 2022), Canada (Treasury Board of Canada Secretariat, 2021), and Japan (Ministry of Economy, Trade, and Industry, 2021). Our main contributions, therefore, are to provide an overview of *The Roadmap* to those unfamiliar with its content and comment on our key takeaways listed above. Our intended readerships are those with an interest in the UK AI strategy and those with an interest in AI ethics' effect on regulation and industry.

## Overview

*The Roadmap* is comprised of five major sections, namely:

1. *Introduction*: the document begins by setting out its high-level strategic goal, which is to balance the opportunity that AI offers with a credible system of governance. All with a view to developing trust which enables AI adoption. Assigning accountability to AI systems and having AI regulation without getting “trustworthy information on how it is used” have been identified as current problems with the creation of trustworthy AI governance. *The Roadmap* sets out how these problems will be addressed.
2. *The Role of AI Assurance:* the document sets out the nature of assurance, the purpose of which is to create evidence-based confidence in AI systems. This includes outlining the relationship between assurance and trust and how this applied to AI development and adoption. This includes such things as the role of AI audits and risk assessments.
3. *Roadmap to a Mature AI Assurance Ecosystem:* here the CDEI proposes a vision of what a mature AI assurance ecosystem should look like.
4. *A Mature Ecosystem Requires Ongoing Effort:* here the CDEI outlines the tensions and limits of AI assurance as well as how these issues can be managed.
5. *The CDEI's Next Steps:* here the steps needed to build a mature AI assurance ecosystem are outlined and how the CDEI plans to implement these.

In this section, we provide an overview of the extended version of *The Roadmap* (CDEI, 2021), following the layout above. Within each subsection, we have reproduced the contents and each section's subtitles in the overview, with respective sections condensed. Where we quote *The Roadmap*, we provide the section in which this can be found in parenthesized italics.

### Introduction

*The Roadmap* begins by outlining its broad strategic goal which is to provide a vision of a mature AI assurance ecosystem in the UK. AI presents invaluable opportunities to industry, but it occasions many novel and significant risks that reduce trust in its adoption. It is this gap that assurance is meant to address. Within the introduction, we identify five key themes:

- *Opportunity: The Roadmap* begins with the CDEI envisaging “data-driven technologies” bringing significant benefits to society. Examples cited include the ability to improve the efficiency of existing processes such as supply chain management; the creation of new tools for analysis and decision-making; DeepMind's protein folding breakthrough; and the potential for operating a green energy grid.
- *Risks: The Roadmap* also states that risks are associated with the adoption of AI systems. Because of the autonomous nature of AI, with machine learning being stated in particular, it is difficult to assign accountability to AI systems when they cause harm and their complexity makes them difficult for affected parties to understand.
- *Scalability:* Developing frameworks for governance is just as complex as the complexity of AI systems, particularly when working across different national jurisdictions and social contexts.
- *Governance:* As AI technologies become more commonplace, tools will be needed to assess an AI system's trustworthiness. For this, an assurance service industry for AI is envisioned, similar to that of the accounting and cybersecurity industries. *The Roadmap* states that “It is not enough to set out standards and rules about how we expect AI systems to be used. It is also important that we have trustworthy information about whether they are following those rules” (*Why we need AI assuranc*e). AI assurance is proposed to play a key role within AI governance by ensuring accurate information is created on the adherence to future AI regulations. Assurance is also important for compliance with other relevant regulations, and this includes the creation of risk assessments, for example, assessing the “fairness” of a particular AI system. The AI assurance ecosystem could emulate the development of the professional services and cybersecurity industries while having the potential to be just as beneficial to the UK economy.
- *Trust:* Consumer trust is vital for the widespread adoption of AI systems. It is envisioned that AI assurance (we elaborate on this term in Section The role of AI assurance) would play a key part in enabling “both trust in and the trustworthiness of AI systems” (*Why we need AI assurance*). Without proper AI assurance, a system cannot be fully trusted to bring benefits without causing unexpected harm. An effective AI assurance ecosystem will mean that, through the use of assurance tools as well as regulations, a sufficient amount of evidence would be created for users of AI systems to form genuine trust in them.

The Roadmap is split into two sections. Section Introduction surveys the establishment and role of a mature AI assurance ecosystem and how it would be used for the creation of trustworthy AI. This section is covered in this review in Section The role of AI assurance.

Section Overview outlines how an AI assurance ecosystem should develop. Six priority areas to make the current AI assurance ecosystem into a mature one are identified (an overview of these points is provided in Section Roadmap to a mature AI assurance ecosystem). This section is covered in this review in Sections Roadmap to a mature AI assurance ecosystem, A mature ecosystem requires ongoing effort, and The CDEI's next steps.

### The role of AI assurance

Given the need for systemic trust outlined in the previous section, *The Roadmap* continues with the specifics of AI assurance and why it is needed to address this problem. This section outlines the kind of trust building that AI assurance should aim to achieve, drawing on examples from other industries.

- *Trust Facilitates Adoption*: If organizations have more trust in AI systems, they would be more likely to adopt them. Untrustworthy AI systems may cause “reputational damage and public backlash” (*What is AI assurance and why do we need it?*). *The Roadmap* asserts that there is currently an insufficient amount of information and specialist knowledge to check claims about AI trustworthiness.
- *Trust/Trustworthiness: The Roadmap* makes a distinction between trust and trustworthiness. “Misplaced trust” is described as “unfounded trust”: “Someone might trust something, even if it is not in fact trustworthy” (*Trust and trustworthiness*). Trustworthiness is stated to be if something can be relied upon. “When we talk about trustworthiness, we mean whether something is deserving of people's trust” (*Trust and trustworthiness*). A trustworthy AI system or organization may not be fully trusted by its users, and this would be unjustified mistrust.
  ° *Justified Trust:* The review states that an AI assurance ecosystem would be able to build justified trust (both trust and trustworthiness) in AI systems through collecting and evaluating information on AI systems to provide evidence of systems being either trustworthy or untrustworthy.
  ° *Communication:* Assurance engagements will allow an organization to communicate claims on whether an AI system is deemed reliable and trustworthy or the opposite.
- *Assurance Legacy Industries:* The review shows how elements of other assurance industries can be used for AI systems. The Roadmap provides examples from the accounting profession.
- *AI Auditing Techniques*: The Roadmap gives examples of auditing techniques that are used for AI systems. The first three have been summarized as follows:
  ° Impact assessments—Used to assess the effect of an AI system or policy may have on its stakeholders as well as how a system or policy may be affected by regulation.
  ° Impact evaluation—Assessing the effect of an AI system or policy after it has been implemented.
  ° Risk assessment—Identifying the potential risks involved when deploying AI.
- *Services:* The review states that AI assurance services are “distinctive and important” (*The role of assurance in AI governance*), but just one part of AI governance.
- *Border Ecosystem (regulation, standards, etc.):* AI governance is influenced by all who are involved in AI. Other aspects of AI governance—i.e., aside from assurance services—include regulation and standards. An AI assurance ecosystem would ensure AI systems could be assessed and verified against regulation and other criteria. An AI assurance ecosystem would be able to offer an “agile ‘regulatory market' of assurance services” (*The role of assurance in AI governance*). This would include both for-profit and not-for-profit organizations which would operate in a way to support regulators and ultimately manage risk without hindering innovation.
- *Compliance: One* of the AI assurance's main jobs is to ensure AI systems comply with regulation. *The Roadmap* states that this would involve interpreting regulation for specific circumstances and providing the technical expertise necessary to keep AI systems compliant to regulation for a system's life cycle.
- *(Export) Market:* Providing AI assurance would also be important for the export market by helping the creators of AI systems comply with international regulations. AI assurance would also be key in building stakeholder trust, especially when an AI system does not have suitable regulation.
- *Agents: The Roadmap* highlights that many actors would be involved in an AI assurance ecosystem. These range from those involved directly in the AI supply chain, assurance providers themselves, and the government. Other important actors would include academic researchers, journalists, accreditation/service providers, and those affected by the use of AI systems. Each actor plays “a number of interdependent roles” (*Roles and responsibilities in the AI assurance ecosystem*) for demonstrating trust and the creation of trustworthy AI systems.

### Roadmap to a mature AI assurance ecosystem

Achieving widespread trust in AI is a matter of facilitating an ecosystem of trust, with assurance at its center. This section outlines the nature of such an ecosystem and presents the key challenges to its construction. Specifically, here *The Roadmap* highlights the importance of assurance for AI in creating justified trust as well as reducing “unjustified mistrust”. This would ensure organizations could deploy AI systems with minimal harm to people, property, and society. Further, it asserts that an AI assurance ecosystem will involve the input of multiple stakeholders, each with their own perspectives, skills, and tools. Indeed, the development of the mature ecosystem could not solely rely on the technical characteristics of AI systems but must also rely upon “subject matter expertise” beyond technical assessment (*Roadmap to a mature AI assurance ecosystem*) for how and where such systems are to be used. While the AI assurance ecosystem defined by the CDEI already exists and “contains the right ingredients for success” (*Roadmap to a mature AI assurance ecosystem*), it is considered highly fragmented. The market for AI assurance has started to grow naturally, but the CDEI sees it as needing to be shaped to respond to “the full spectrum of risks and compliance issues presented by AI systems”.

*The Roadmap*, therefore, details the six areas where the current AI assurance ecosystem needs to develop in order to become mature:

- ∙ Generating demand for assurance:
  ° The reputation of businesses is identified as the key reason for the early demand of AI assurance. Public awareness and interest from regulators is also identified, and this is in response to high-profile failures that have driven the desire for accountability when developing and using AI-related systems.

British companies building AI systems for the export market often have to adhere to foreign AI regulations and will have to continue to do so as more countries introduce regulation. *The Roadmap* identifies the EU AI regulations and Canadian AIA as examples of AI-related regulations that the UK's export market already has to adhere to or prepare for in the near future.

° The CDEI predicts that in a mature AI ecosystem, demand for assurance will be driven by; the need to know that systems or processes work reliably; keeping customer and staff trust in the systems they deploy; addressing the potential risk that may come with an AI system; adherence to regulation; and “competing on the basis of public trust”.
  ■ A risk of “ethics washing” (CDEI, 2021) is identified in the current AI assurance ecosystem. This is where AI is audited selectively to benefit an organization's reputation. This is recognized as a potential hindrance for creating trustworthy AI systems. A better understanding on how the AI supply chain should approach accountability is “required to drive demand for assurance”.
° Supporting demand for assurance:
  ■ *The Market for AI Assurance Services:* As demand for AI assurance grows, the market for AI assurance services will have to develop to accommodate more actors in the AI supply chain. This is because the CDEI expects assurance to become increasingly time-consuming and complex to remain part of the AI supply chain. The CDEI sees assurance being offered either as a separate service or as a “specialist in-house capacity”, similar to model risk management in the finance industry. External assurance providers are expected to meet the increase in demand. These providers will include existing professional services firms as well as “specialized start-ups”. AI assurance will have to be independently verified to ensure that justified trust is formed, and business conflict of interest is avoided.
  ■ On top of new assurance techniques and regulation, existing regulation and assurance will have to adapt to AI-related issues. Other AI-related assurance services include testbeds, currently used for the testing of autonomous vehicles. The ICO has begun producing initiatives to ensure AI systems are used and developed in a trustworthy manner. This includes the AI auditing framework and draft guidance (Arslan, 2020).
° Developing standards:
  ■ Standards are described by *The Roadmap* as the “crucial enablers of AI assurance”. *The Roadmap* views standards as being able to provide a reliable basis for people to “share the same expectations” (*Standards and assurance enablers*). The CDEI wants to, as described previously, emulate the success of the cybersecurity industry, and the UK has the potential to play a leading role in the creation of international standards. To create truly trustworthy AI, standards must also analyze the potential social and ethical impact of AI; however, technical AI standards would also support future assurance efforts.
° The role of professionalization and specialized skills:
  ■ *The Roadmap* states that the AI assurance profession will grow as the demand for third-party accreditation increases. Accreditation may initially come from master's degrees and vocational courses. In the future, an accreditation body may be the next step, similar to the Institute for Chartered Accountants in England and Wales. No existing model for professional accreditation currently exists for AI as it is a “multi-disciplinary” practice. *The Roadmap* proposes that the United Kingdom Accreditation Service (UKAS), the UK's national accreditation body, and the British Computer Society could “partner together” to have the expertise for accreditation in AI assurance.
° The role of regulation:
  ■ “An effective assurance ecosystem is key to effective regulation in many areas”. *The Roadmap* states that AI assurance and regulation complement each other. An effective AI assurance ecosystem would free up regulators' limited resources to focus on “high-risk, contentious, or novel areas”, while the AI assurance ecosystem ensures “good practice”, meaning AI assurance services would make sure organizations adhere to regulations. Regulators would help in setting the expectations for how AI assurance will be conducted and create the “supporting structure” for how the ecosystem would develop. Many regulators have already begun developing AI guidelines specifically for their sector, and some have published guidelines.
° The role of independent researchers:
  ■ As the AI assurance ecosystem develops and becomes more widespread, the role of independent researchers is likely to become more important. Academic research has already become key in the identification of untrustworthy AI. Most notably with bias in AI services. *The Roadmap* sees future independent researchers “highlighting untrustworthy development and gaps in regulatory regimes”. Cybersecurity infrastructure is an example of independent researchers having a successful impact on an industry. This occurs through the identification of vulnerabilities through schemes like “bug bounties”. Also, academic researchers and other stakeholders, such as journalists and activists, are expected to play an important role in providing “scrutiny and transparency”.

### A mature ecosystem requires ongoing effort

As the “ecosystem” metaphor suggests, systemic trust is a dynamic and evolving phenomenon. This section surveys the ongoing effort needed to sustain the system.

° *Interdependence:* In order to create a mature AI ecosystem, those involved are dependent on each other's expertise and “co-evolve”, while also having competing interests.
° *Five Tensions:* The CDEI has identified five key tensions in the AI assurance ecosystem:
  ° Regulators want specific rules, but the government does not want to enact new laws; this could be because the regulation may fall “beyond the scope of the state” (The roadmap to an effective AI assurance ecosystem, 2021) or not be set too prematurely or be informed by “popular sentiment” rather than careful consideration.
  ° There is a trade-off between “risk minimization and encouraging innovation”.
  ° Who would accept responsibility for good AI systems? The *Roadmap* highlights the tension between developers and executives as the party accountable for how well an AI system runs.
  ° The issue of gaming. This is when openness in a system can render it liable to being changed in a manner that superficially solves a particular concern, where in fact transparency has facilitated this “tweaking” strategy.
  ° How can an organization communicate trust (effectively) to third parties, where the primary concern is that those who manage relevant systems may find the imperative is to provide a meaningful explanation that makes sense to the affected party (in a manner that does not simply appeal to technical jargon).
  ° “A balanced approach” is necessary to ensure AI systems are safely adopted without affecting developers.
• *Limits to Assurance:* There are limits to assurance. *The Roadmap* identifies the gaps between the current AI assurance ecosystem and a future mature ecosystem.
  ° *IP/Access:* Intellectual property rights may cause difficulty in carrying out external audits without sacrificing trade secrets. Assurance services have to have sufficient access to AI while protecting intellectual property. Privacy concerns when auditing datasets could be a potential difficulty for AI assurance. When auditing an AI system, sensitive data may be exposed.
  ° *Standardization:* There are limits to the standardization of AI systems. It is highly unlikely that all forms of AI assurance could be standardized, nor could the AI systems.

### The CDEI's next steps

Finally, *The Roadmap* outlines the immediate tasks necessary to creating the ecosystem:

To create a mature AI assurance ecosystem, they will actively work with its partners to support the trustworthiness of AI innovation. To enable trustworthy assurance practices, the CDEI will publish an AI assurance guide, focused on the delivery of AI assurance. The CDEI will work with partners to develop standardization and support effective regulation and policy. Furthermore, the CDEI will work with existing accreditation bodies with the aim of creating accreditation of the AI assurance ecosystem. They also plan to have an advising, supporting, and influencing role in the ecosystem, working with other government organizations, professional, and industry bodies to develop on projects such as the AI standards hub as well as promoting standardization, government policy, and the responsible adoption of AI.

Convening and consensus building is a crucial next step for the CDEI to bring the currently fragmented efforts around AI assurance together. They will do this by working with stakeholders and developing solutions to the “blockers to AI assurance” and accelerating the development of the AI assurance ecosystem.

## Commentary

In this section, first we select some key themes identified in *The Roadmap* and offer targeted commentary. Below we expand on each of these themes, drawing on content from the roadmap and contextualizing it within the wider AI ethics and assurance movements.

Second, we abstract more specific concerns, such as accreditation, technical standardization, and industry transparency.

### Building justified trust

One of the challenges identified with creating a trustworthy AI assurance ecosystem is the lack of universally accepted standards for AI development. This is something that is addressed in *The Roadmap* Indeed, in January 2022, the Department for Digital, Culture, Media, and Sport (DCMS), alongside the Office of Artificial Intelligence, announced an initiative to develop standards for AI technologies with the intention of establishing standards that would be globally recognized. The Alan Turing Institute has been selected to lead this new AI Standards Hub, which will create tools and educational material to aid businesses and other organizations with the adoption of UK-led global standards. This will be done in an effort to “put the UK at the forefront of this rapidly developing area” (New UK initiative to shape global standards for Artificial Intelligence, 2022). If the UK were to succeed in creating internationally recognized standards, it could be a significant benefit to future British AI assurance providers. However, to increase the social and commercial benefits of AI, organizations must be given the incentive to protect their reputation through effective assurance initiatives. These should be based upon accepted standards and regulation to dissuade rather than relying on public relations campaigns, being selective of auditing only where there is the threat of public scrutiny and internal, unaccountable AI ethics standards.

#### Comment

It is recognized that to create justified trust for AI systems, certification must be created and adopted to reliably verify that an AI system's risk has been mitigated as well as provide evidence that work by the assurer has been done correctly. However, this is considered a long-term goal for the CDEI, while AI assurance services are both offered and used in the present day. AI assurance providers currently have the ability to provide “implicit accreditation” of AI assurance, meaning they effectively assume the risk and responsibility for an AI system if it were to fail. In the short term, a solution is needed to share risk between both the assurer and the organization developing the AI system. An example of certification found in another industry is the International Information System Security Certification Consortium, or (ISC)^2^, offers the Certified Information Systems Security Professional (CISSP) (Cybersecurity Certification| CISSP - Certified Information Systems Security Professional | (ISC)^2^, 2022), an internationally recognized certification for cybersecurity practitioners. However, we note that the development reliable certification for AI practitioners is likely to take time due to the technology's recent adoption.

Further, we raise a point of contention in relation to *The Roadmap's* analogies to cybersecurity and accounting when referring to successful assurance industries and practices. Specifically, we feel that these do not adequately address the complexity and novel nature of AI assurance. Effectively communicating trust to multiple stakeholders without oversimplifying important information is likely to be a significant difficulty for AI assurance. Such information will need to be communicated throughout the AI supply chain to assurance providers, the government and the general public. Both accounting and cybersecurity are mature industries with established regulation, certification, and standards; however, they are not as broad in scale as AI assurance. *The Roadmap's* approach to assuring AI systems through certification and standards, similar to existing assurance industries, does not recognize the speed of innovation in AI development, and this means that standards and certification may be overtaken by newer technologies. Standards are being developed for creating trustworthy AI assurance both in the UK and internationally (ISO/IEC JTC 1/SC 42—Artificial intelligence, 2017), but these have long timeframes and so may not be as effective at reducing harm in the near future as AI development practices change.

### The role of research

Research, conducted by both academics and “independent researchers”, is highlighted in *The Roadmap* as having an important role in increasing coordination between the academic world of AI assurance research and the young AI assurance industry. However, the current difficulties with creating such a system include:

- *Industry transparency:*
  Companies developing AI systems often conduct open-source research; however, transparency with production systems has much less transparency. This could make assuring AI systems in production more difficult or less effective.
- *Policies for research:*
  Understanding what kind of policies will be needed for AI research to be conducted in a safe and ethical way. We will also highlight the research standards found in academia.

#### Comment

Much like in academia, it is not uncommon for researchers of AI to make their findings and code open access to encourage reproducibility and collaboration between researchers. This would make the development of AI assurance research a simpler process. However, if companies are apprehensive about sharing data or systems, this could lead to difficulty doing applied research. Presently, there are no obligations for private sector algorithmic transparency (Kazim et al., 2021). For researchers and industry to collaborate successfully, it is our view that industry transparency would either have to be mandated or there would need to be an increased culture of transparency between research organizations and the private sector. Ideally, the AI systems used by an organization would be as open and transparent as the AI found in research but this is implausible as such systems are often key to revenue generation and are protected by intellectual property regulations. While some attempts to provide some transparency have been made by organizations, such statements are often issued after the use of such systems has come under scrutiny from the government or the public. An example of this is the Explainability Statement published by HireVue (HireVue AI Explainability Statement, 2022) after the Electronic Privacy Information Center (EPIC) filed a complaint with the Federal Trade Commission (FTC) stating that the company “purports to evaluate a job applicant's qualifications based upon their appearance by means of an opaque, proprietary algorithm” (In re HireVue EPIC, 2019).

Another barrier for successful industry and research collaboration is the lack of ethical procedures in AI research presently. For example, the fields of psychology (Ethical principles of psychologists code of conduct, 2017) or medicine (Jiang et al., 2017; Code of Medical Ethics Overview, 2022; The British Medical Association, 2022) have stringent research ethics guidelines, something that is not seen with AI research and that has been recognized in *The Roadmap*. In response to the development of more trustworthy AI, ethics guidelines have been developed by the Alan Turing Institute (Leslie, 2019) and the European Union (Ethics Guidelines for Trustworthy AI, 2018) to ensure that AI ethics are embedded at the development stage of a system and to build public trust in AI.

### Current developments in AI assurance within industry

Artificial intelligence is already being widely used across society, by both the private and public sectors. Its prevalence has led to existing processes being made more efficient (Bhadoria et al., 2021) and the creation of new tools. The use of AI systems has led to success in many industries. A notable example is healthcare, where machine learning is used in early detection, diagnosis, and treatment of neurology and cardiology (Jiang et al., 2017). However, there have also been some high-profile failures such as Amazon's recruitment tool that was biased against female applicants (Dastin, 2018) and Microsoft's “Tay” chatbot that posted offensive tweets based on user interactions (Vincent, 2016). Controversies like these have led to increased public and government interest in the adoption of methods to regulate the use and development of AI. Indeed, recent years have seen progress toward a consensus on the need for assurance in the creation and adoption of AI systems. To this end, we have started to see the creation of industry standards, consideration of AI ethics (Kazim and Koshiyama, 2021), and the creation of AI practitioners.

#### Comment

*The Roadmap* states that “standards are the crucial enablers of AI assurance” (The roadmap to an effective AI assurance ecosystem—extended version, 2021). Technical standards are necessary for the development of an effective AI assurance ecosystem and can help to ensure that companies can complete effective compliance audits to predetermined standards. Currently, many of the standards used for software validation and verification would be inadequate for the development of AI systems. This is due AI's ability to learn, relearn, and adapt autonomously; errors may “manifest themselves” (Batarseh et al., 2021) without being specifically coded. The complexity of assuring AI systems becomes more apparent when examining the different areas of AI, and first, there are many different AI areas, such as neural networks and machine learning, each with different techniques for assuring. The way data are collected and modeled and the sample size also influence the outcome of an AI model which will also require assurance. Bias is a possible complication that can occur and can begin during data collection (Batarseh et al., 2021). For example, a facial recognition model could be exposed to an adversarial input, to mimic genuine data, thus making a system less robust. Additional assurance for a system would be needed to mitigate such risks (Batarseh et al., 2021).

Technical standardization for the management of risk in AI development is currently being developed but is not yet accepted by the development community. Examples of standards in development include the Institute of Electrical and Electronics Engineer (IEEE), IEEE 7000-2021 standard—Addressing Ethical Concerns During Systems Design (IEEE SA—IEEE 7000-2021, 2021). This standard will give businesses “a system engineering standard approach integrating human and social values into traditional systems engineering design”. The International Organisation for Standardisation's Joint Technical Committee (ISO/IEC JTC 1) is also developing standards for the development of artificial intelligence (ISO/IEC JTC 1/SC 42—Artificial intelligence, 2017). Further, education programs have emerged to create and educate AI assurance practitioners, such as the master's AI Ethics and Society, delivered by the University of Cambridge and the Leverhulme Centre for the Future of Intelligence (MSt in AI Ethics Society, 2021).

### International regulation for AI

Artificial intelligence governance is viewed as an important part of many proposals for the regulation of AI development found around the world. *The Roadmap* emphasizes the need for conformity with other regulations in order to facilitate trade. The UK's approach to AI assurance has similarities and differences to that of other legal frameworks, which we discuss in this section.

The European Union (EU) has proposed the *Artificial Intelligence Act* (hereafter “EU AI Act”) (European Commission, 2021). This legislation aims to reduce and monitor the creation of potentially risky AI systems by categorizing AI systems and practices according to the level of risk they potentially pose to their users. These categories are as follows:

- Prohibited Artificial Intelligence practices:
  These are systems that produce an unacceptable level of risk to its users and are considered contrary to the values of the EU. Prohibited systems and practices include those which infringe upon European citizen's rights, systems that have the potential to manipulate or exploit vulnerable groups and cause physical or psychological harm, social scoring systems for “public authorities”, and, in most cases, “real-time” remote biometric identification systems in public spaces.
- High-Risk AI Systems:
  Systems deemed high risk by the artificial intelligence act are subject to compliance procedures in order to be deemed safe for use in the EU. Such systems include: AI systems involving biometrics, the management of critical infrastructure, access to education or employment, access to “essential private services”, as well as law enforcement and border control.
- Low- or Minimal-Risk AI Systems:
  Systems deemed “low or minimal risk” are permitted without restriction; however, developers of these systems will be encouraged to follow a code of conduct based upon the requirements of high-risk systems. An example of a “low- or minimal-risk” AI system would be spam filters.

The EU AI Act is one of the most significant examples of AI assurance regulation and one that may be significant in the development of future AI regulation. The AI act takes a harm reduction-based approach that would affect AI development based primarily on the risk generated by the system and any deployed system would have to adhere to the legislation including for research. While *The Roadmap* has not specifically any regulation for research, it views the contribution of research to AI assurance as an important part of the ecosystem.

Like the EU, the United States of America has regulatory proposals at federal level, in addition to state and local governance of AI systems. At the federal level is the *Algorithmic Accountability Act* (Wyden et al., 2022), which requires companies that use algorithms and other “automated decision systems” to “conduct impact assessments for bias, effectiveness, and other factors, when using automated decision systems to make critical decisions”. The aim of this is to reduce the implementation of AI systems that are intentionally or unintentionally discriminatory as well as mitigating the potential harm of poorly designed algorithms. This bill would apply to companies that have “$50,000,000 in average annual gross receipts” and more than one million users. An example of state-level governance is the Illinois Artificial Intelligence Video Interview Act (JD Supra, 2022), which came into effect on January 1, 2020 (820 ILCS 42/Artificial Intelligence Video Interview Act., 2020). The legislation, which requires employers to notify each applicant individually that their interview will be analyzed by an AI system, affects organizations that are hiring for positions based in the state and use AI systems to analyze recorded video interviews. Applicants are also asked to consent to the use of the system when this notice is given, and there are limitations placed on who the videos can be shared with (“persons whose expertise or technology is necessary in order to evaluate an applicant's fitness for a position”) (820 ILCS 42/Artificial Intelligence Video Interview Act., 2020). Employers and third parties are also required to delete an applicant's interview on request and data on the race and ethnicity of applicants who are “not afforded the opportunity for an in-person interview after the use of artificial intelligence analysis” and “the race and ethnicity of applicants who are hired”. These data must be submitted to the Illinois Department of Commerce and Economic Opportunity annually.

At the local level, the New York City Council (The New York City Council—File : Int 1894-2020, 2020) has passed a bill that would require organizations using AI-based hiring tools to conduct annual bias audits and disclose how these systems will be used in the hiring process publicly (Lai, 2021). Candidates will also be able to “request an alternative selection process”. Organizations found using biased or undisclosed AI hiring systems face a maximum fine of $1,500 for every violation. This legislation will come into force from January 1, 2023.

This legislation indicates that AI systems used for decision-making in the hiring process are coming under intense scrutiny and along with legislation from New York City (discussed below) a precedent may emerge from these laws that could be used in different legislations or become the basis for a standard adopted by organizations using AI around the world, including ones based in the UK. But, only in local legislation, it marks a significant step in the recognition of AI governance and the need for an AI assurance industry that could carry out these annual bias audits. This is also an example of where the CDEI envisages a UK AI assurance company could export to in the future. The AI regulations from the United States are very much aligned with the values of federalism, meaning that local governments can implement regulation on systems when they see fit. A disadvantage to this would be that these regulations could lead to confusion and contradiction with other AI regulations across state or other local boundaries.

Beyond the major jurisdictions of the UK, EU, and USA, Japan's Ministry of Economy, Trade, and Industry (METI) published guidelines on implementing AI principles in July 2021 “how AI principles should be implemented” (Ministry of Economy, Trade, and Industry, 2021). This report looks at what standards, guidelines, and regulations may be necessary for Japan while also taking similar AI policies found abroad into account, including references to the EU AI Act and the OECD. The report was written with the input of an “expert group” from law, academia, and industry. Much like *The Roadmap*, METI's report is an analysis of the types of assurance necessary to minimize the risk of deploying unsafe AI systems while also adhering to international regulation and standards. The report does not explicitly propose new regulations for Japan.

Going beyond just governance, the Canadian government has developed an applied approach to AI assurance, releasing its *Algorithmic Impact Assessment* tool (Treasury Board of Canada Secretariat, 2021) for the use of policymakers and other officials to assess and mitigate the risks associated with deploying AI systems. This impact assessment is designed to score an AI system based on “areas of risk”. These areas include the source of data, the type of data, the motivation for introducing an AI system into the decision-making process, the transparency of the algorithm, and the ease of explaining its use. While the *Algorithmic Impact Assessment* tool is not a proposal for future regulation, it offers a solution to help the government test the impact an AI system may have before it is deployed. It follows a risk-based approach for AI governance. Similar tools could be introduced in the future for the private sector in Canada and in other countries.

#### Comment

With many nations proposing AI-specific legislation for the development and use of AI systems, the true impact they will have on organizations is not yet fully understood. For example, while New York City has passed legislation mandating bias audits of automated employment decision tools (^*^cite legislation^*^), this requirement only applies to employers hiring applicants within city limits, but we expect this to have wider-reaching consequences, where employers will also choose to audit algorithms not used within the city (Hilliard, 2022). Further, the EU AI Act could have a potentially global effect on the future of Algorithmic Accountability and how AI systems are developed. Being a larger market than New York City alone, organizations will have to comply with the regulation if the systems effect citizen in the EU. The EU AI may have a similar effect that GDPR had to data protection, where it became the global standard despite only being enforced within the EU (Li et al., 2019).

## Conclusion

Artificial intelligence ethics is an important field and is one that is gaining traction as the risks of using AI are realized and increasingly researched. One of the major ways to address concerns about the use of AI is to increase the governance of its use and determine best practices for both industry and research, which is the goal of the CDEI's roadmap to an effective AI assurance ecosystem (CDEI, 2021). Specifically, the UK's approach to AI assurance is based on creating an ecosystem of trust, thus embedding assurance practices and making an AI assurance industry a key part of the AI supply chain. The roadmap seeks to do this by (i) increasing justified trust of AI by creating an AI standards hub and inviting the development of accreditation for auditors; (ii) promoting collaboration between industry and academic research to establish best practices; (iii) calling for the creation of widely adopted technical standards; and (iv) converging with global regulation attempting to govern the use of AI. While the UK's approach appears to be more “pro-innovation”, it is currently unknown what effect the export market will have on the development of AI products. We therefore look forward to seeing what measures will be taken to ensure this industry develops to be as effective at creating trustworthy AI systems, with special regard to the adoption of AI assurance standards with the creation of an AI Standards Hub (Enabling trustworthy innovation to thrive in the UK—Centre for Data Ethics and Innovation Blog., 2021) and developing AI assurance practitioners.

## Author contributions

EB, EK, AH, and MT were responsible for the conceptualization and writing—original draft. SZ and AK were responsible for review and editing. All authors contributed to the article and approved the submitted version.

## Conflict of interest

Authors EB, EK, MT, SZ, AH, and AK were employed by Holistic AI.

## Publisher's note

All claims expressed in this article are solely those of the authors and do not necessarily represent those of their affiliated organizations, or those of the publisher, the editors and the reviewers. Any product that may be evaluated in this article, or claim that may be made by its manufacturer, is not guaranteed or endorsed by the publisher.

## References

[B1] 820 ILCS 42/Artificial Intelligence Video Interview Act. (2020). Available online at: https://www.ilga.gov/legislation/ilcs/ilcs3.asp?ActID=4015&ChapterID=68 (accessed April 5, 2022).

[B2] Arslan A. K. (2020). A design framework for auditing AI. JMEST 7, 12768–12776. Available online at: https://www.jmest.org/wp-content/uploads/JMESTN42353353.pdf (accessed March 22, 2022).35157476

[B3] Batarseh F. A. Freeman L. Huang C.-H. (2021). A survey on artificial intelligence assurance. J. Big Data 8, 60. 10.1186/s40537-021-00445-7

[B4] Bhadoria R. S. Pandey M. K. Kundu P. (2021). RVFR: random vector forest regression model for integrated & enhanced approach in forest fires predictions. Ecol. Inform. 66, 101471. 10.1016/j.ecoinf.2021.101471

[B5] CDEI (2021). The Roadmap to An Effective AI Assurance Ecosystem - Extended Version. GOV.UK. Available online at: https://www.gov.uk/government/publications/the-roadmap-to-an-effective-ai-assurance-ecosystem/the-roadmap-to-an-effective-ai-assurance-ecosystem-extended-version (accessed March 1, 2022).

[B6] Code of Medical Ethics Overview (2022). American Medical Association. Available online at: https://www.ama-assn.org/delivering-care/ethics/code-medical-ethics-overview (accessed April 8, 2022).

[B7] Cybersecurity Certification| CISSP - Certified Information Systems Security Professional | (ISC)2 (2022). Available online at: https://www.isc2.org:443/Certifications/CISSP (accessed April 5, 2022).

[B8] Dastin J. (2018). Amazon scraps secret AI recruiting tool that showed bias against women. Reuters. Available online at: https://www.reuters.com/article/us-amazon-com-jobs-automation-insight-idUSKCN1MK08G (accessed April 8, 2022).

[B9] Enabling trustworthy innovation to thrive in the UK—Centre for Data Ethics Innovation Blog (2021). Available online at: https://cdei.blog.gov.uk/2021/09/10/enabling-trustworthy-innovation-to-thrive-in-the-uk/ (accessed April 11, 2022).

[B10] Ethical principles of psychologists code of conduct (2017). Available online at: https://www.apa.org/ethics/code (accessed April 8, 2022).

[B11] Ethics Guidelines for Trustworthy AI (2018). FUTURIUM - European Commission. Available online at: https://ec.europa.eu/futurium/en/ai-alliance-consultation (accessed March 29, 2022).

[B12] European Commission (2021). Proposal for a Regulation of the European Parliament and of the Council Laying Down Harmonised Rules on Artificial Intelligence (Artificial Intelligence Act) and Amending Certain Union Legislative Acts. Available online at: https://eur-lex.europa.eu/legal-content/EN/TXT/?uri=CELEX%3A52021PC0206 (accessed March 1, 2022).

[B13] Freeman L. Batarseh F. A. Kuhn D. R. Raunak M. S. Kacker R. N. (2022). The path to a consensus on artificial intelligence assurance. Computer 55, 82–86. 10.1109/MC.2021.312902733667591

[B14] Hilliard A. (2022). Regulating the robots: NYC mandates “bias audits” for AI-driven employment decisions - IFOW. The Institute for the Future of Work. Available online at: https://www.ifow.org/news-articles/regulating-robots-nyc-bias-audits-ai-driven-employment-decisions (accessed June 17, 2022).

[B15] HireVue AI Explainability Statement (2022). Available online at: https://www.hirevue.com/press-release/hirevue-launches-ai-explainability-statement-in-hr-industry-first (accessed April 11, 2022).

[B16] IEEE SA—IEEE 7000-2021 (2021). SA Main Site. Available online at: https://standards.ieee.org/ieee/7000/6781/ (accessed April 11, 2022).

[B17] In re HireVue EPIC (2019). Electronic Privacy Information Center. Available online at: https://epic.org/documents/in-re-hirevue/ (accessed April 8, 2022).

[B18] ISO/IEC JTC 1/SC 42—Artificial intelligence. (2017). ISO. Available online at: https://www.iso.org/cms/render/live/en/sites/isoorg/contents/data/committee/67/94/6794475.html (accessed April 11, 2022).

[B19] JD Supra (2022). Employers Using AI in Hiring Take Note: Illinois' Artificial Intelligence Video Interview Act Is Now in Effect. Available online at: https://www.jdsupra.com/legalnews/employers-using-ai-in-hiring-take-note-54767/ (accessed April 8, 2022).

[B20] Jiang F. Jiang Y. Zhi H. Dong Y. Li H. Ma S.. (2017). Artificial intelligence in healthcare: past, present and future. Stroke Vasc. Neurol. 2, 230–243. 10.1136/svn-2017-000101 29507784 PMC5829945

[B21] Kazim E. Almeida D. Kingsman N. Kerrigan C. Koshiyama A. Lomas E.. (2021). Innovation and opportunity: review of the UK's national AI strategy. Discov. Artif. Intell. 1, 14. 10.1007/s44163-021-00014-0

[B22] Kazim E. Koshiyama A. (2020). AI assurance processes. SSRN J. 1-99. 10.2139/ssrn.3685087

[B23] Kazim E. Koshiyama A. S. (2021). A high-level overview of AI ethics. Patterns 2, 100314. 10.1016/j.patter.2021.100314 34553166 PMC8441585

[B24] Lai N. T. L. (2021). Why New York City is cracking down on AI in hiring. *Brookings*. Available online at: https://www.brookings.edu/blog/techtank/2021/12/20/why-new-york-city-is-cracking-down-on-ai-in-hiring/ (accessed April 5 2022).

[B25] Leslie D. (2019). Understanding artificial intelligence ethics and safety: a guide for the responsible design and implementation of AI systems in the public sector. Zenodo. 10.2139/ssrn.3403301

[B26] Li H. Yu L. He W. (2019). The impact of GDPR on global technology development. J. Glob. Inform. Technol. Manage. 22, 1–6. 10.1080/1097198X.2019.1569186

[B27] Ministry of Economy Trade Industry. (2021). AI Governance in Japan Ver. 1.1 Report From the Expert Group on How AI Principles Should Be Implemented. Available online at: https://www.meti.go.jp/shingikai/mono_info_service/ai_shakai_jisso/pdf/20210709_8.pdf (accessed March 29, 2022).

[B28] MSt in AI Ethics Society (2021). Available online at: https://www.ice.cam.ac.uk/course/mst-artificial-intelligence-ethics-and-society (accessed April 8, 2022).

[B29] National AI Strategy—HTML version (2021). Available online at: https://www.gov.uk/government/publications/national-ai-strategy/national-ai-strategy-html-version (accessed March 1, 2022).

[B30] National Data Strategy (2020). Available online at: https://www.gov.uk/government/publications/uk-national-data-strategy/national-data-strategy (accessed March 8, 2022).

[B31] New UK initiative to shape global standards for Artificial Intelligence (2022). Available online at: https://www.gov.uk/government/news/new-uk-initiative-to-shape-global-standards-for-artificial-intelligence (accessed March 25, 2022).

[B32] PricewaterhouseCoopers (2017). The Economic Impact of Artificial Intelligence on the UK Economy. Available online at: https://www.pwc.co.uk/economic-services/assets/ai-uk-report-v2.pdf (accessed June 22, 2022).

[B33] The British Medical Association (2022). Ethics The British Medical Association is the trade union and professional body for doctors in the UK. Available at: https://www.bma.org.uk/advice-and-support/ethics (accesse*d Apri*l 8, 2022).

[B34] The need for effective AI assurance—Centre for Data Ethics Innovation Blog (2021). Available online at: https://cdei.blog.gov.uk/2021/04/15/the-need-for-effective-ai-assurance/ (accessed March 21, 2022).

[B35] The New York City Council—File #: Int 1894-2020 (2020). Available online at: https://legistar.council.nyc.gov/LegislationDetail.aspx?ID=4344524&GUID=B051915D-A9AC-451E-81F8-6596032FA3F9&Options=Advanced&Search (accessed April 5, 2022).

[B36] The roadmap to an effective AI assurance ecosystem (2021). Available online at: https://www.gov.uk/government/publications/the-roadmap-to-an-effective-ai-assurance-ecosystem/the-roadmap-to-an-effective-ai-assurance-ecosystem (accessed March 21, 2022).

[B37] The roadmap to an effective AI assurance ecosystem—extended version (2021). Available online at: https://www.gov.uk/government/publications/the-roadmap-to-an-effective-ai-assurance-ecosystem/the-roadmap-to-an-effective-ai-assurance-ecosystem-extended-version (accessed March 1, 2022).

[B38] Treasury Board of Canada Secretariat (2021). Algorithmic Impact Assessment Tool. Available online at: https://www.canada.ca/en/government/system/digital-government/digital-government-innovations/responsible-use-ai/algorithmic-impact-assessment.html (accessed March 2, 2022).

[B39] Vincent J. (2016). Twitter taught Microsoft's friendly AI chatbot to be a racist asshole in less than a day. The Verge. Available online at: https://www.theverge.com/2016/3/24/11297050/tay-microsoft-chatbot-racist (accessed April 8 2022).

[B40] Wyden R. Booker C. Clarke Y. (2022). Algorithmic Accountability Act of 2022. Available online at: https://www.wyden.senate.gov/download/algorithmic-accountability-act-of-2022-bill-text (accessed March 02, 2022).

